# N-cadherin is Key to Expression of the Nucleus Pulposus Cell Phenotype under Selective Substrate Culture Conditions

**DOI:** 10.1038/srep28038

**Published:** 2016-06-13

**Authors:** Priscilla Y Hwang, Liufang Jing, Jun Chen, Foon-Lian Lim, Ruhang Tang, Hyowon Choi, Kenneth M Cheung, Makarand V Risbud, Charles A Gersbach, Farshid Guilak, Victor Y Leung, Lori A Setton

**Affiliations:** 1 Department of Biomedical Engineering, Duke University, Durham NC 27713, USA; 2 Department of Biomedical Engineering, Washington University in St. Louis, St. Louis Missouri 63130, USA; 3 Department of Orthopaedic Surgery, Duke University Medical Center, Durham NC 27713, USA; 4 Departments of Orthopaedics & Traumatology, The University of Hong Kong, Hong Kong SAR, China; 5 Department of Orthopaedic Surgery, Washington University in St. Louis, St. Louis Missouri 63110, USA; 6 Department of Orthopaedic Surgery, Thomas Jefferson University, Philadelphia PA, USA

## Abstract

Nucleus pulposus (NP) cells of the intervertebral disc are essential for synthesizing extracellular matrix that contributes to disc health and mechanical function. NP cells have a unique morphology and molecular expression pattern derived from their notochordal origin, and reside in N-cadherin (CDH2) positive cell clusters *in vivo*. With disc degeneration, NP cells undergo morphologic and phenotypic changes including loss of CDH2 expression and ability to form cell clusters. Here, we investigate the role of CDH2 positive cell clusters in preserving healthy, biosynthetically active NP cells. Using a laminin-functionalized hydrogel system designed to mimic features of the native NP microenvironment, we demonstrate NP cell phenotype and morphology is preserved only when NP cells form CDH2 positive cell clusters. Knockdown (CRISPRi) or blocking CDH2 expression *in vitro* and *in vivo* results in loss of a healthy NP cell. Findings also reveal that degenerate human NP cells that are CDH2 negative can be promoted to re-express CDH2 and healthy, juvenile NP matrix synthesis patterns by promoting cell clustering for controlled microenvironment conditions. This work also identifies CDH2 interactions with β-catenin-regulated signaling as one mechanism by which CDH2-mediated cell interactions can control NP cell phenotype and biosynthesis towards maintenance of healthy intervertebral disc tissues.

Disorders of the intervertebral disc (IVD) contribute to pain and disability in affected individuals, such that low back and neck pain are ranked as the top contributors to global burden of disease^1^,^2^. Much work has been done to understand the biological and anatomical changes associated with disc disorders and aging-related degeneration, such as loss of disc height and hydration, diminished blood supply in the endplates, and anulus fibrosus tears^3^,^4^. Consensus suggests that changes in the nucleus pulposus (NP) region of the IVD, such as decreased cellularity, water content and loss of proteoglycan content in the extracellular matrix (ECM) are amongst the earliest events leading to disc degeneration^3^,^5^,^6^. Cells of the NP region are largely responsible for producing functional ECM and secreting chemokines and growth factors that regulate matrix synthesis in the healthy, hydrated, and mechanically-functional IVD^7^,^8^,^9^. The observed loss of NP cellularity and changes in NP cell phenotype are thus believed to be key regulators of the onset and progression of disc degeneration.

Healthy, juvenile NP cells are remnants of the embryonic notochord^10^,^11^, and are characterized as large, vacuolated cells^12^,^13^,^14^ that are capable of forming cell clusters^15^,^16^,^17^ within their native ECM^18^,^19^,^20^. Gene and protein analysis of human^21^, bovine^22^, porcine, and rat^23^ NP tissue has identified the presence of several laminin isoforms and N-cadherin (CDH2) in healthy, juvenile tissues. With disc degeneration or aging, NP cells transition to a sparse population of small, chondrocyte-like cells that lose their ability to form cell-cell interactions, with decreased to no expression of CDH2 (Fig. 1)^13^,^18^,^19^,^21^,^24^. Coincident with these changes in NP cell number and morphology are ECM changes that include a stiffening of the ECM^25^,^26^ and loss of laminin expression^14^,^27^,^28^. In other cell types, CDHs regulate an assortment of cell behaviors and phenotype, and ablation or perturbation of CDH-mediated cell adhesions result in developmental abnormalities and pathological processes^29^,^30^. CDH2 is key for normal gastrulation and neural crest development^31^,^32^, regulates cell-cell interactions during mesenchymal condensation in chondrogenesis^33^,^34^, and plays an essential role during myogenesis and myotube formation^35^. Recent consensus has identified a panel of markers specific to the healthy juvenile NP cell phenotype, including CDH2, transcriptional factors (e.g., brachyury), matrix-related (e.g., proteoglycan, type II collagen) and cell signaling molecules (e.g., sonic hedgehog)^36^,^37^,^38^,^39^,^40^, and changes in expression for these markers is associated with degeneration^23^,^24^,^41^. We hypothesize that CDH2 positive (CDH2+) cells and CDH2-mediated cell contacts in the juvenile NP cell are features necessary for preserving the key markers of the healthy, NP-specific cell phenotype and morphology.

The objective of this work was to investigate the role of CDH2-mediated cell contacts in regulating human NP cell morphology and phenotype. We used an *in vitro* hydrogel system composed of laminin and polyethylene glycol (PEG) as a model of the juvenile NP microenvironment^14^,^27^,^42^,^43^,^44^. Juvenile porcine NP cells were studied for their ability to retain features of the NP notochordal origin *in vitro*^8^,^13^, while adult degenerate and juvenile human NP cells were studied to test for an ability of controlled culture conditions to promote re-expression of healthy NP-specific cell features. Loss-of-CDH2-function studies (CRISPRi or blocking antibodies) were performed to evaluate a role for CDH2 in regulating porcine NP cell behavior *in vitro*. A role for CDH2 loss in contributing to IVD degeneration was also evaluated in a rat model following intradiscal injection of CDH2 function-blocking antibodies. Degenerate, human NP cells, which are CDH2 negative, were also cultured on our laminin hydrogel model system to test if microenvironmental cues could promote CDH2 expression and revert degenerate cells towards a juvenile phenotype. Results indicate juvenile human and porcine NP cells form CDH2+ cell clusters when cultured on soft laminin hydrogels, with patterns for biosynthesis and molecular expression consistent with the healthy, juvenile NP phenotype. Blocking or knocking-down CDH2 in NP cells results in decreased cell clustering ability, reduced matrix biosynthesis and a loss of features of the juvenile NP cell *in vitro* and *in vivo*. Altogether, our findings demonstrate that CDH2 plays a critical role in both juvenile and adult degenerate NP cells to promote features of a healthy and biosynthetically active NP cell.

## Results

### NP cells form CDH2-positive cell clusters on soft laminin hydrogels, with associated elevated matrix synthesis and gene expression for NP-specific markers

Previously, we demonstrated the ability for porcine cells to form cell clusters (a feature of juvenile NP cells) *in vitro* by culturing upon polymerized Matrigel (basement membrane extract) or upon polyacrylamide gels of <0.7 kPa stiffness functionalized with Matrigel and other matrix proteins^45^. In this study, we cultured NP cells on laminin-functionalized polyethylene (PEG-LM) hydrogels designed to be “soft” (0.3 kPa) or “stiff” (1.2 kPa) in order to achieve more precise control of hydrogel stiffness and presentation of laminin proteins (Supplemental Figure 1). Formulations of PEG-LM deemed “soft” (0.3 kPa) were suitable for supporting NP cell cluster formation, as previously observed for polymerized Matrigel^17^ or polyacrylamide gels^45^.

**Figure 2:**
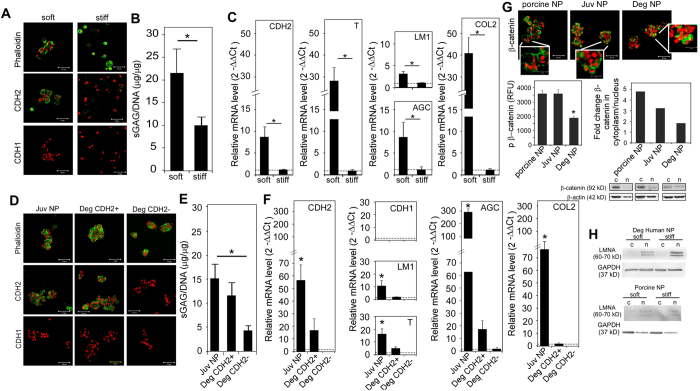
Porcine and human NP cells form CDH2-positive (+) cell clusters on soft, laminin (LM) hydrogels with preservation of juvenile NP phenotype. (**A**) Representative immunostaining of phalloidin and cadherins for porcine NP cells on soft and stiff PEG-LM (green = protein, red = cell nuclei, scale bar = 50 μm). (**B**) Changes in sGAG production for porcine NP cells on soft and stiff PEG-LM. (**C**) Quantification of gene expression for juvenile NP cell phenotype markers in porcine NP cells on soft, relative to NP cells on stiff (CDH2 = N-cadherin, T = brachyury, LM1 = Laminin1, AGC = aggrecan, COL2 = type II collagen). (**D**) Representative immunostaining of phalloidin and cadherins for juvenile (juv) and degenerate (deg) human NP cells on soft PEG-LM (green = protein, red = cell nuclei, scale bar = 50 μm, CDH2 + = CDH2 positive, CDH2− = CDH2 negative). (**E**) Same as B but with human NP cells on PEG-LM. (**F**) Same as C but with human NP cells on PEG-LM (additional marker CDH1 was quantified in human). (**G**) Representative immunostaining for β-catenin (green) in NP cells on soft PEG-LM with associated changes in phosphorylated β-catenin, fold-change for β-catenin in the cytoplasm to nucleus, with corresponding western blot images for β-catenin (immunostaining images scale bar = 50 μm, with higher magnification inset scale bar = 20 μm). (**H**) Western blot images for LMNA expression on soft and stiff PEG-LM (All western blot images were cropped to display protein expression concisely; see Supplemental Figure 4 for full western blots) (For all studies: 2-way ANOVA with Tukey’s post-hoc analysis, *p < 0.05).

Juvenile NP cells (porcine and human) cultured on soft, PEG-LM maintained a rounded morphology and formed clusters associated with positive CDH2, not CDH1, expression (Fig. 2A,D). By comparison, juvenile NP cells did not form cell clusters on stiff PEG-LM hydrogels (1.2 kPa, Fig. 2A for porcine, human not shown), indicating the importance of matrix stiffness and ECM ligand presentation in regulating NP cell behavior. Correspondingly, changes in cell phenotype were also observed for NP cells cultured on different PEG-LM environments: higher levels of sulfated glycosaminoglycan (sGAG) synthesis and gene expression of relevant NP markers (CDH2, brachyury, laminin, aggrecan, and type II collagen) were observed for NP cells cultured on soft, PEG-LM compared to stiff PEG-LM (Fig. 2B–F, *p < 0.05). To further confirm that the observed outputs are unique to NP cellular responses, we also cultured AF cells on PEG-LM. In general, AF cell behavior and phenotype were not affected by PEG-LM: AF cells remained as individual cells with no significant differences in matrix synthesis or gene expression for most of the investigated markers (Supplemental Figure 2). One exception was elevated gene expression for type II collagen for porcine, but not human, AF cells upon soft, PEG-LM. Together these findings verify soft, laminin-rich environments are appropriate for supporting features specific to healthy, juvenile NP cells.

Additionally, we sought to determine if these controlled culture conditions would support degenerate human NP cells to re-express features of a healthy, juvenile NP phenotype. In particular, we evaluated re-expression of CDH2 in degenerate NP cells as prior studies have revealed negative immunostaining for CDH2^46^ in adult degenerate, but not juvenile NP tissue sections. To answer this question, we cultured degenerate human IVD cells on PEG-LM hydrogels and evaluated changes in cell phenotype. Culturing degenerate human NP cells, regardless of age, resulted in cell cluster formation on soft PEG-LM (Fig. 2D); some degenerate NP cells were positive for CDH2 under these conditions while others were not (Fig. 2D). Following this observation, we separated degenerate human NP cells into groups classified as CDH2 positive (>90% cells positive via immunostaining) and CDH2 negative (<90%) (representative images shown in Fig. 2D). Both CDH2 positive and CDH2 negative cells were capable of forming cell clusters as shown in Fig. 2D; this unexpected finding clearly demonstrates that multiple cell-cell interactions may be involved in the formation of cell clusters in the human. Nevertheless, we found significantly higher levels of sGAG synthesis and gene expression of NP-specific markers for samples that were CDH2+ compared to samples that were CDH2- (Fig. 2E,F, *p < 0.05). These results provide strong evidence for a relationship between CDH2 and the expression of juvenile NP-specific features, even for degenerated human NP cells.

### β-catenin and associated signaling in NP cells on soft PEG-LM

In many cell types, CDH2 associates directly with β-catenin that is then tethered to the cell membrane^30^. When tethered to the cell membrane, β-catenin is unable to translocate to the nucleus and activate a panel of downstream signaling events that can regulate cell phenotype^47^,^48^. We sought to determine if the relationship between CDH2 and juvenile NP cell phenotype was due to interactions between CDH2 and β-catenin that acted to prevent specific signaling events in NP cells. We observed β-catenin localization at the cell membrane in porcine and juvenile human NP cells when cultured on soft PEG-LM hydrogels, with a pattern similar to that of CDH2 localization (Fig. 2G). For degenerate human NP cells, β-catenin expression was diffusely distributed throughout the cell instead of localizing to the cell membrane. This difference in β-catenin expression suggests β-catenin turnover in the degenerate human NP cell that could be due to changes in β-catenin phosphorylation and/or translocation to the nucleus^49^,^50^. Immunostaining findings prompted us to further investigate β-catenin activity in NP cells by quantifying protein expression for phosphorylated β-catenin (pβ-cat) and β-catenin translocation to the nucleus. Significantly lower levels of pβ-cat were quantified in degenerate human NP cells compared to juvenile human or porcine NP cells (Fig. 2G, *p < 0.05). After separating the nuclear and cytoplasmic fractions, we found equal levels of β-catenin protein expression in the nucleus and cytoplasm for degenerate NP cells, whereas healthy, juvenile NP cells had higher levels of β-catenin in the cytoplasm (Fig. 2G). These observations suggest that β-catenin translocates to the nucleus in degenerate human NP cells, but remains tethered to the cell membrane in the presence of CDH2 for the juvenile NP cells. While further studies are needed to confirm downstream cellular mechanisms and behavior, our analysis of β-catenin protein expression in NP cells suggest a relationship between CDH2 and β-catenin that may control NP cell phenotype.

Apart from tethering to CDH2, β-catenin has also been shown to interact with lamin A/C (LMNA), a fibrillar protein of the nuclear envelope^51^,^52^. In human mesenchymal stem cells (MSCs), overexpression of LMNA facilitated β-catenin entry into the cell nucleus and led to increased levels of nuclear β-catenin^51^ that impacted MSC differentiation. Thus, we hypothesized that higher levels of LMNA would be observed in degenerate NP cells under soft culture conditions since higher levels of β-catenin translocation were observed in degenerate NP cells for this case. Indeed, protein analysis of LMNA (Fig. 2H) revealed higher expression of LMNA in degenerate human NP cells when cultured upon cluster-promoting, soft PEG-LM hydrogels, as compared to non-cluster-promoting stiff PEG-LM. All together, our studies demonstrate differences in β-catenin and LMNA for degenerate human NP cells when allowed to form cell clusters, which is consistent with promotion of CDH2 expression and its contribution to expression of juvenile NP-specific behaviors and phenotype for soft PEG-LM culture conditions.

### CDH2 knockdown via CRISPR-dCas9 prevents NP cell clustering on soft PEG-LM and results in loss of juvenile NP cell phenotype and morphology

**Figure 3:**
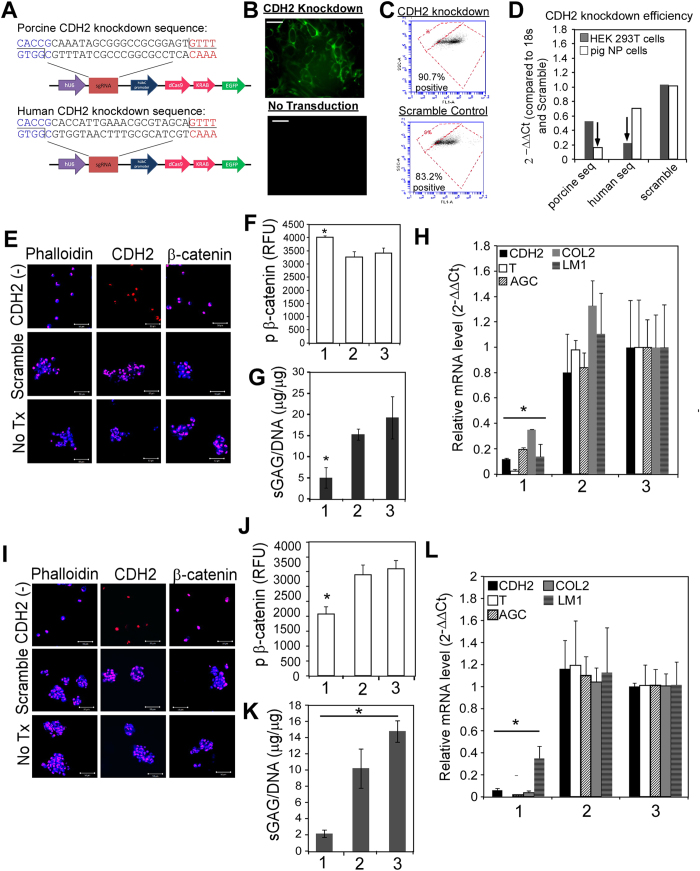
CDH2 knockdown (via CRISPRi) prevents cell cluster formation on soft, PEG-LM in porcine and human NP cells, with associated loss of juvenile NP phenotype. (**A**) CRISPRi construct sequence. (**B**) Representative images of successful CDH2 knockdown analyzed by fluorescence microscopy. (**C**) Validation of successful CDH2 knockdown via flow cytometry. (**D**) Quantification of CDH2 knockdown via qRT-PCR; arrows indicate successful CDH2 knockdown using species-specific CRISPRs (porcine seq = porcine sequence, human seq = human sequence). (**E**) Representative immunostaining for phalloidin, CDH2 and β-catenin in porcine NP cells after CDH2 knockdown, compared to scramble control and no treatment (blue = protein, pink/red = cell nuclei, scale bar = 50 μm). (**F**) Quantification of total phosphorylated β-catenin levels in porcine NP cells (1 = CDH2 (−), 2 = scramble, 3 = no treatment). (**G**) Changes in sGAG in CDH2 knockdown porcine NP cells on soft. (**H**) Changes in gene expression for CDH2 knockdown porcine NP cells on soft, compared to stiff (CDH2 = N-cadherin, T = brachyury, LM1 = laminin, COL2 = type II collagen, AGC = aggrecan; * denotes that all genes are significantly different from scramble and no treatment controls) (**I–L**) same as (**E**–**H**) respectively, but with CDH2 knockdon in juvenile human NP cells (For all studies: 2-way ANOVA with Tukey’s post-hoc analysis, *p < 0.05).

We performed CDH2 knockdown studies using CRISPRi to confirm that matrix production and gene expression of NP-specific markers were activated downstream of CDH2-mediated cell-cell contact formation. We designed a CDH2 CRISPRi using dCas9-KRAB-GFP with 20 base pair sequences from the promoter regions of porcine and human CDH2 (Fig. 3A) and verified their ability to knockdown CDH2 expression via fluorescence imaging, flow cytometry, and gene expression (Fig. 3B–D). We transduced porcine NP cells, and juvenile human NP cells that have not yet lost their CDH2 expression, with our CDH2 CRISPRi. When cultured on soft PEG-LM, we found that CDH2 knockdown prevented NP cells from forming cell clusters (Fig. 3E,I); instead, NP cells remained as individual cells that were negative for CDH2 expression (Fig. 3E,I). Corresponding to the loss of cell cluster formation, CDH2 knockdown in NP cells resulted in significantly lower levels of sGAG production and gene expression for all NP markers analyzed (Fig. 3G,H and K,L). Next, we determined changes in β-catenin signaling by quantifying phosphorylated β-catenin levels after CDH2 knockdown. Knockdown of CDH2 in porcine NP cells resulted in significantly higher levels of pβ-cat compared to non-transduced NP cells, signifying higher levels of cytoplasmic β-catenin (Fig. 3F, *p < 0.05). This unexpected result that β-catenin continues to be activated with significant reduction in CDH2 suggests that multiple cell-receptor and cell-cell interactions may be regulating β-catenin activity in the porcine juvenile cell. Conversely, knockdown of CDH2 in juvenile human NP cells resulted in significantly lower levels of pβ-cat compared to non-transduced NP cells (Fig. 2J, *p < 0.05), which may signify faster pβ-cat turnover, either via translocation to the nucleus or degradation by the cell. All together, these results corroborate the role for CDH2-mediated regulation of the β-catenin signaling pathway in the NP cell and suggest CDH2-mediated regulation of β-catenin may be substantive in the human NP cell.

### Blocking antibody treatment establishes regulatory role of CDH2, not CDH1, in regulating NP cell phenotype and morphology

**Figure 4:**
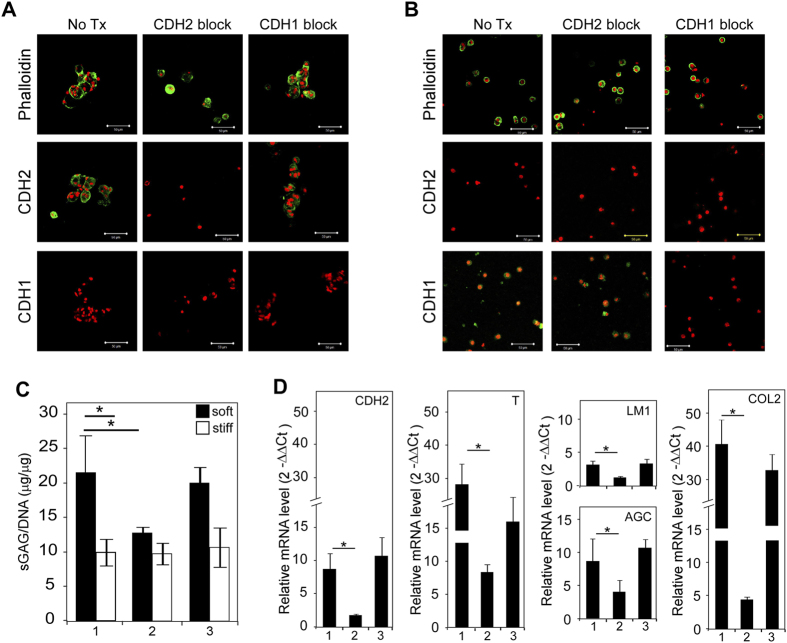
CDH2, not CDH1, blocking antibody treatment prevents porcine NP cells from forming cell clusters on soft PEG-LM, along with associated loss of juvenile NP cell phenotype features. (**A,B**) Representative immunostaining of phalloidin, CDH2, and CDH1 for NP and AF cells, respectively (green = protein, red = cell nuclei, scale bar = 50 μm). (**C**) Changes in sGAG production for porcine NP cells on soft and stiff PEG-LM after blocking antibody treatment. (**D**) Quantification of gene expression for juvenile NP cell phenotype markers in porcine NP cells on soft, relative to NP cells on stiff after blocking antibody treatment (2-way ANOVA with Tukey’s post-hoc analysis, *p < 0.05) (Key: 1 = No treatment condition, 2 = CDH2 blocking antibody treatment, 3 = CDH1 blocking antibody treatment; CDH2 = N-cadherin, T = brachyury, LM1 = Laminin1, AGC = aggrecan, COL2 = type II collagen).

To confirm the role of CDH2 in regulating NP cell phenotype and morphology, we performed *in vitro* studies following delivery of CDH function-blocking antibodies to porcine NP cells. We cultured porcine NP cells on soft PEG-LM and supplemented media with CDH2 or CDH1 blocking antibody. Similar to CDH2 CRISPRi knockdown studies, a decreased ability to form cell clusters was observed in NP cells when CDH2 was blocked; instead, cells remained as individual cells (Fig. 4A) behaving in a similar fashion as AF cells cultured on PEG-LM (Fig. 4B). Correspondingly, decreased levels of sGAG and gene expression for NP-specific markers were also observed in NP cells after CDH2 blocking antibody treatment (Fig. 4C,D). In contrast, delivery of the CDH1 function-blocking antibody to NP cells on soft PEG-LM did not alter cell phenotype and morphology. NP cells continued to form cell clusters with the ability to synthesize matrix and preserve gene expression of juvenile NP markers on soft PEG-LM (Fig. 3A,C,D, *p < 0.05), which confirms that NP cell behavior is CDH2 mediated. These findings reveal a unique role of CDH2 in regulating NP cell phenotype and morphology, which cannot be compensated by CDH1.

In addition to revealing the unique role of CDH2 in regulating NP cell behavior, we also wanted to confirm that these findings are unique to this cell type. Therefore, we also treated neighboring AF cells with CDH function-blocking antibodies. Regardless of CDH blocking antibody treatment, AF cells remained as individual cells on PEG-LM hydrogels in a similar manner to no treatment conditions (Fig. 4B). As expected, no significant differences were observed for matrix production or gene expression of CDH2, T, laminin, and aggrecan compared to no treatment conditions (Supplemental Figure 3); these results suggest CDHs do not regulate AF cell behavior on PEG-LM in the same manner as NP cells.

### *In vivo* blocking of CDH2 leads to loss of juvenile NP cell phenotype features

**Figure 5:**
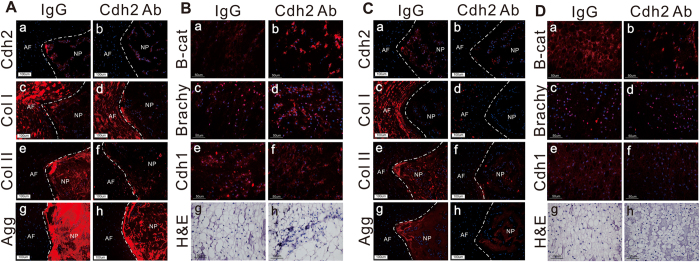
Delivering CDH2 blocking antibody *in vivo* results in loss of juvenile NP cell phenotype. **(A)** Representative immunostaining of CDH2 (a,b), type I collagen (Col I) (c,d), type II collagen (Col II) (e,f), and aggrecan (agg) (g,h) in rat tail discs 2 weeks after intradiscal injection of CDH2 blocking antibody (Cdh2 Ab) or control immunoglobulin (IgG) (red = protein, blue = cell nuclei, scale bar = 100 μm, NP = nucleus pulposus; AF = anulus fibrosus). (**B)** Representative immunostaining of non-phosphorylated β-catenin (a,b), brachyury (c,d), and E-cadherin (e,f) and representative histological assessment of H&E staining (g,h) 2 weeks after intradiscal injection of Cdh2 Ab or IgG (red = protein, blue = cell nuclei, scale bar = 50 μm, NP tissue only). **(C,D)** Similar to panel A and B, but assessed 8 weeks after blocking antibody delivery.

We chose to further test the suspected relationship between CDH2 loss and degenerated NP cells and tissue with an *in vivo* model of disc degeneration in the rat. Intradiscal delivery of CDH2 blocking antibodies or IgG controls was performed for rat tail discs. Two weeks after injection, decreased type II collagen and aggrecan expression were observed in the NP region of discs receiving CDH2 blocking antibodies as compared to IgG controls (Fig. 5A). Additionally, β-catenin was no longer tethered to the cellular membrane, as evidenced by increased β-catenin expression in the cytoplasmic region of NP cells (Fig. 5B). With time, we continued to observe a loss of juvenile NP cell features in segments receiving the CDH2 blocking antibodies. Eight weeks after CDH2 blocking antibody injection, type II collagen and aggrecan expression continued to decrease in the NP region (Fig. 5C), along with decreased expression of brachyury, when compared to both IgG control and two-week samples. Along with decreased expression of these juvenile NP-specific markers, we also observed a decreased number of cell-cell contacts (Fig. 5, CDH1, CDH2, and hemotoxylin/eosin stain) in the NP region of the disc. The continual reduction in protein expression of juvenile NP cell features along with decreased cell-cell contacts following CDH2 blocking antibody treatment leads us to believe that NP cells do not have a mechanism to re-express CDH2 once its function has been disturbed, which could be a contributing factor to disc degeneration.

In neighboring AF tissue, we did not observe significant differences in the markers analyzed two weeks after CDH2 blocking antibody treatment compared to IgG control (Fig. 5A,B). It was not until eight weeks after CDH2 blocking antibody treatment that we observed decreased type I collagen expression in AF tissue compared to IgG control (Fig. 5C). Overall, our *in vivo* findings validate *in vitro* findings that CDH2 is necessary for maintaining the expression of juvenile NP extracellular matrix, and that CDH2 interactions with β-catenin are one factor regulating cell signaling events in the juvenile NP cell downstream of CDH2 engagement.

## Discussion

The results of this study reveal the importance of CDH2-mediated cell contacts in preserving features of the juvenile NP cell phenotype. Using a soft PEG-LM hydrogel as a system to mimic features of native NP ECM, porcine and human juvenile NP cells were able to form CDH2+ cell clusters with associated higher levels of matrix synthesis and gene expression of NP-specific markers; in contrast, culturing both cell types upon stiff PEG-LM was unable to reproduce cell cluster formation and associated matrix synthesis and NP-specific marker expression. Cellular signaling studies reveal a role for β-catenin and laminA in contributing to cell signaling events that may regulate the NP-specific cell phenotype. Additionally, blocking or knocking down CDH2 in juvenile NP cells *in vitro* resulted in decreased ability for cells to form cell-cell contacts or retain features of the juvenile cell. These *in vitro* findings were confirmed in our *in vivo* studies where we delivered CDH2 blocking antibody to the NP region of the disc. Furthermore, we revealed the potential to revert degenerate NP cells back towards a CDH2+ juvenile phenotype by culturing degenerate, human NP cells in the soft PEG-LM culture system. Overall, this study indicates that CDH2 plays a prominent role in preserving features of the juvenile NP cell phenotype and that environmental cues of stiffness and ligand presentation are potent regulators of that molecular expression pattern and cell morphology. The knowledge gained from our work can be used to inform biotechnology and therapies to manipulate cell phenotype for the treatment of disc degeneration and related pathologies.

A novel finding in this study is the ability to not only maintain the juvenile NP cell phenotype for juvenile NP cells *in vitro*, but also the potential to promote degenerate NP cells to adopt the expression pattern of juvenile NP phenotype on soft PEG-LM. Culturing some degenerate, human NP cells on soft PEG-LM promoted formation of CDH2+ cell contacts, and levels of cellular biosynthesis that were significantly higher than culturing the same cells on stiff PEG-LM. While CDH2- degenerate NP cells were still able to form cell contacts on soft, PEG-LM, these cell clusters were not CDH2 mediated, and were not able to preserve juvenile NP cell features; all CDH2- degenerate NP cells had matrix synthesis and gene expression patterns similar to culturing on stiff PEG-LM. These results reveal the integral role of CDH2 in regulating juvenile NP cell morphology and phenotype. Prior studies demonstrated the importance of ECM stiffness and ligand presentation in dictating NP cell behaviors but did not investigate the role of cell-cell adhesion molecules during this phenomenon^14^,^17^,^19^,^27^,^45^. AFM testing of cell stiffness establish porcine NP cells have elasticities between 0.3 and 0.8 kPa^53^ and NP cells naturally reside in soft, gelatinous ECM with elasticities ranging from 0.3–5 kPa^25^,^26^,^54^,^55^, suggesting that the soft culture conditions were an appropriate mimic of the elasticity of the healthy, juvenile ECM^45^,^53^. It remains unclear, however, why some degenerate NP cells were able to form CDH2+ cell clusters on these soft PEG-LM culture surfaces whereas others are not. Nevertheless, work in other cell types has shown that microenvironmental cues can regulate CDH expression and activity, as we have shown here for NP cells, and thereby influence cell fate^56^,^57^. Indeed, our observations of CDH2 expression and NP cell fate in the degenerate human NP cells are consistent with this concept.

In this study, we used CRISPR/dCas9 (clustered regularly interspaced short palindromic repeats) to knockdown CDH2 by targeting genomic sites of the CDH2 promoter in NP cells^58^. CRISPR genome editing methods are easy to use, highly versatile, and have been successfully applied in many model organisms and immortalized cell systems^59^. In primary cells such as the porcine NP cells studied here, there has been limited success with commercially available genome editing technologies. By using dCas9 as the packaging system, we were able to knockdown CDH2 expression with fewer off-target effects as compared to use of blocking antibodies^60^, and we were able to achieve very high levels of knockdown. Findings that CDH2 knockdown via CRISPR/dCas9 results in loss of CDH2-mediated cell-cell contacts and loss of juvenile NP cell features corroborate with our CDH2 blocking antibody findings that CDH2 is the primary CDH regulating juvenile NP cell phenotype and morphology during NP cell cluster formation.

Our study is the first to deliver CDH2 blocking antibody *in vivo* to the NP region of the intervertebral disc. The *in vivo* work supports our *in vitro* findings that CDH2 has a unique cell adhesive function found only in NP cells and substantiates the role of CDH2 in promoting maintenance of healthy NP cells and associated NP-specific ECM. Specifically, we found that blocking CDH2 resulted in decreased expression of many key ECM markers that are characteristic of healthy NP tissue. Prior studies of NP tissue engineered constructs^43^,^61^ have also demonstrated that CDH2 was consistently elevated in constructs that possessed the most favorable outcomes, specifically enhanced matrix synthesis and cell survival.

Findings that AF cell clustering behaviors are not affected by culture conditions or CDH blocking antibody treatment illustrates important differences between AF and NP cells of the same source and age. In prior work, porcine AF cells were shown to form cell clusters upon polymerized Matrigel, and cluster formation was disrupted by incubation with CDH1 but not CDH2 blocking antibody treatment. The difference between the current work with culture upon PEG-LM and this prior work using polymerized Matrigel suggest a role for an alternate ECM ligand in regulating AF cell behaviors . Indeed, AF tissue has a very different cellular and matrix composition from NP tissue^62^, and is characterized as highly organized and stiff (compressive modulus: 0.12–2.3 MPa) tissue with layers of collagenous lamellae^7^. Additionally, during disc development, cell alignment is necessary for successful transition to AF cells and matrix production, indicating a need for a template for AF cell deposition^15^,^63^. For this reason, much work has focused on development of scaffolds that can promote AF cell alignment using materials such as collagen, fibronectin, PCL, alginate or hybrid scaffolds^63^,^64^. The results of the current study corroborate these prior observations that rounded AF cell attachment and interaction with laminin is neither necessary nor critical to preservation of the AF cell phenotype, in comparison to cells of the NP region^15^. In future studies, we hope to overexpress CDH2 in AF cells to further assess the unique role of CDH2.

Wnt signaling regulates functions in multiple cell types, principally during embryonic development, cell differentiation, and cell proliferation^49^,^50^,^65^. β-catenin regulates canonical Wnt signaling and may be one mechanism by which CDH2 expression and engagement contributes to regulated biosynthesis and gene expression in cells of the NP. Indeed, results of the current study are the first study to link changes in CDH2 expression with changes in β-catenin in NP cells. Our findings reveal β-catenin was mainly localized to the cell membrane in juvenile human and porcine NP cells, with higher levels of β-catenin translocation to the nucleus quantified in degenerate human NP cells. Additionally, significantly higher levels of phosphorylated β-catenin was quantified in juvenile NP cells compared to degenerate NP cells. These findings are consistent with the proposed role for β-catenin in promoting transcriptional activation that contributes to a shift in cell molecular expression patterns and phenotypes in degenerate NP cells. In NP cells, β-catenin/Wnt signaling has been reported to suppress cell proliferation and induce cell senescence, suggesting its involvement in IVD degeneration^66^. The observed translocation of β-catenin to the nucleus in the absence of positive CDH2 expression in adult degenerate nucleus pulposus cells, and decreased protein expression in rat motion segments receiving a CDH2 blocking antibody may be consistent with an activation of β-catenin under degenerated disc conditions. However, additional studies confirming downstream signaling events for β-catenin in promoting transcription activation would be necessary to confirm this suggestion.

Previously, we demonstrated the ability for porcine NP cells to form cell clusters (a feature of juvenile NP cells) *in vitro* by culturing upon polymerized Matrigel (basement membrane extract)^17^. We further demonstrated an inability to promote cell cluster formation for juvenile NP cells upon polyacrylamide surfaces functionalized with type II collagen, for surface stiffness over a very broad range from 0.2–12 kPa^45^. Given the specificity of the cellular interaction with laminin, we cultured NP cells on a laminin-functionalized polyethylene glycol (PEG-LM) hydrogel in order to achieve more precise control of hydrogel stiffness and presentation of laminin proteins (Supplemental Figure 1).

Findings from our study, along with prior work^17^,^28^,^43^,^45^, indicate the necessity of both CDH2 and extracellular matrix cues (matrix stiffness and ligand presentation) in order to preserve a biosynthetically active juvenile NP cell, leading us to conclude both cell-matrix and cell-cell interactions are necessary for regulating NP cell phenotype and morphology. In many cell types, cadherin-mediated adherens junctions alter cell phenotype through multiple mechanisms, including direct effects of actin cytoskeletal dynamics^67^,^68^. Also similar to our findings, LaminA/C directly modulates mechanosensitive genes and is believed to contribute to cell phenotype and morphology in many cell types^69^. As such, there is much interest in investigating how NP cell interactions with their ECM can induce differences in stress fiber formation, focal adhesion expression, cytoskeletal contractility, F-actin turnover, or other mechanotransductive cues, which can impact cell-cell contact formation, and alter NP cell behavior. Conclusions from such studies would reveal how cell-matrix interactions can be exploited to maintain CDH2 mediated cell contacts that are necessary to preserve healthy, juvenile NP cells, which can be applied for development of cell delivery or tissue engineering strategies to treat disc degeneration.

## Conclusions

This study is the first to report a role for CDH2-mediated cell clustering in maintaining key features of the NP cell phenotype and morphology both *in vitro* and *in vivo*. Culturing porcine and human NP cells on soft, PEG-LM resulted in the formation of CDH2+ cell clusters that were able to synthesize matrix and preserve gene expression for NP-specific markers. Additionally, cell-signaling studies reveal the role of β-catenin and lamin A/C as downstream signaling molecules that interact with CDH2 to regulate cell phenotype. Blocking CDH2 or CRISPRi mediated knockdown of CDH2 in NP cells *in vitro* or *in vivo* resulted in loss of all features of the juvenile NP phenotype. This work demonstrates that microenvironmental cues can regulate retention of, and reversion to a healthy, biosynthetically active juvenile cell phenotype for the NP cell, and further suggests that CDH2 is a key regulatory molecule in that interaction with microenvironment.

## Methods

### IVD cell isolation

NP and AF cells from lumbar spines of young pigs (4–5 months, Nahunta Pork Outlet, Raleigh NC, n = 27 separate isolation pools were generated and pooled to form 9 independent samples; 3 replicate samples used per *in vitro* experiment) were isolated via enzymatic digestion^28^. The following enzymatic digestion method was adopted to allow for optimal re-expression of any cell surface receptors potentially affected by the enzymatic isolation process^14^,^70^. NP cells were isolated from NP tissues via pronase-collagenase enzymatic digestion, then re-suspended in culture media (Ham’s F-12 media (Gibco, Invitrogen) supplemented with 5% FBS (Hyclone, Thermo Scientific), 100 U/mL penicillin (Gibco) and 100 mg/mL streptomycin (Gibco)). Re-suspended NP cells were cultured in sub-confluent monolayers on conditioned media (collected from rat carcinoma cell line, 804 G) coated tissue culture flasks for 1–5 days before use. AF cells were isolated from AF tissues via pronase digestion, followed by collagenase digestion, then re-suspended in the same culture media, as described above, but with 10% FBS. Re-suspended AF cells were cultured in sub-confluent monolayers on 0.1% gelatin-coated tissue culture flasks for 1–5 days before use.

Human IVD tissue was obtained as to-be-discarded surgical waste, from patients undergoing surgery for treatment of degeneration or scoliosis (n = 22, ages 7–70) at Duke University Medical Center with approval of the Institutional Review Board. The collection of these tissues was performed in accordance with protocols for non-human subjects research (45CFR46.102(f), Department of Health and Human Services, USA), and only gender, race, and age was provided at time of collection. The Duke University Institutional Review Board approved this collection of de-identified human tissues as non-human subjects research prior to beginning this experiment. Samples were separated into classfications corresponding to juvenile (<13 years old) and adult, “degenerated” (35–70 years old) populations for the purpose of this study. Regions corresponding to AF and NP tissue were visually separated, and cells were isolated via enzymatic digestion^28^ in the same manner as porcine IVD. Human NP and AF cells were re-suspended in the same culture media as porcine NP cells except supplemented with 10% FBS and cultured in sub-confluent monolayers on tissue-culture treated flasks for 5–10 days before use.

### Laminin-functionalized polyethylene glycol (PEG-LM) hydrogel synthesis

PEG-LM hydrogels were synthesized via a 2-step process, as previously described^43^,^44^. Briefly, full-length laminin-111 (LM, 6 mg/ml, Trevigen) was PEGylated with acrylate-PEG-N-hydroxysuccinimide (Ac-PEG-NHS, MW = 10 kDa, Creative PEGworks). The resulting PEGylated LM conjugate solution was dialyzed to remove unreacted Ac-PEG-NHS, and LM-111 concentration in each PEGylated LM conjugate precursor solution was determined by measuring absorbance at 280 nm. Varying amounts of 8-arm PEG-acrylate (20 kDa, Creative PEGworks; 4 or 20% w/v) was mixed with 500 μg/ml of PEGylated LM conjugate, injected to 8-well chamber slides (Millicell EZslide, Millipore), and polymerized upon exposure to UV light (3–4 mW cm^−2^) in the presence of 0.1% (w/v) photoinitiator (Irgacure 2959, Ciba Specialty Chemicals).

For all cultures, two formulations of hydrogels were synthesized as 4% PEG-LM (soft, 0.3 kPa) and 20% PEG-LM (stiff, 29 kPa). LM distributions were determined via EZblue protein stain (Sigma-Aldrich) and microscopic imaging of the hydrogel surface (Nikon Eclipse E600, Nikon) revealed LM-111 was uniformly distributed across the surface of PEG-LM hydrogels (~60% of surface area at 4x magnification, supplemental Figure 1), regardless of hydrogel stiffness.

### IVD cell culture on PEG-LM

IVD cells (human or porcine) in monolayer culture were detached from the culture surface using 0.025% trypsin/EDTA (Cambrex) and immediately re-suspended in culture media. NP or AF cells (45,000 cells/well, n = 3 per measured variable) were cultured upon each substrate for up to 96 hours (37 ^○^C, 5% CO_2_).

### CDH2 knockdown in porcine or juvenile human NP cells using clustered regularly interspaced short palindromic repeats (CRISPRs)

#### Plasmid construction

The construction of guideRNA-dCas9-KRAB plasmid was performed in two steps^59^. First, the target sequence for the 5′UTR region of the CDH2 gene (for porcine and human) with adjacent PAM sequences (NGG) was identified using the UCSC Genome Browser for porcine genome. Two single-stranded oligos including the predicted target sequence were annealed, phosphorylated, and inserted into the U6 Church backbone (Addgene 53188) using BBsI restriction enzyme site (NEB). Second, the sgRNA was transferred into the lentiviral destination vector containing dCas9-KRAB-GFP (herein referred to as dCas9, Addgene 53191). To assemble the sgRNA constructs into the lentiviral destination vector, the desired regions from the sgRNA constructs were PCR amplified and BSMBI sites were added on to the ends, and ligated to dCas9. The final plasmid was confirmed in 2 ways: (1) test PCR using the sense primer 5′-TCTTGGCTTTATATATCTTGTGGA-3′, and antisense primer 5′-TCTAAGGCCGAGTCTTATGAGCAG-3′ that amplifies across the sgRNA region, and (2) sequence confirmed (Eton Biosciences). A scrambled sequence (5′-CACCGGCACTACCAGAGCTAACTCA-3′, 5′-AAACTGAGTTAGCTCTGGTAGTGCC-3′) was also synthesized in the same manner as a control.

#### Lentiviral production

All lentiviral vectors used in this study are second generation and were produced using standard viral production methods that have been previously described . Briefly, 300,000 HEK293T cells were plated per well of a 6-well plate. After cell attachment overnight, cells were transfected with 4 μg of transfer vector, 1.2 μg of pMD2.G (Addgene 12259) and 3.2 μg psPAX2 (Addgene 12260) via Lipofectamine 2000 Transfection Reagent (Life Technologies). Media was changed 12–14 hours post-transfection, and viral supernatant was collected 24 and 48 hours after media change, passed through a 0.45 μm filter, pooled and concentrated via centrifugation. Concentrated viral particles were stored in −80°C until use.

#### Lentiviral transduction of NP cells

Freshly isolated porcine NP cells were cultured in 804 G-coated 12-well plates (60,000 cells/well) or juvenile human NP cells were cultured in tissue culture treated 12-well plates (60,000 cells/well), and allowed to attach overnight. After cell attachment, cells were cultured overnight in 1 ml culture media supplemented with 75 μl of concentrated LV and 8 μg/mL polybrene (Sigma-Aldrich). Transduction medium was then exchanged for fresh culture media, and cells were cultured for 6 days until use. Percentage of positive cells was determined via flow cytometry (Accuri C6 flow cytometer, Becton Dickinson). qRT-PCR was used to identify the knockdown efficiency of CDH2 (Life Technologies). Cells were then cultured upon PEG-LM (45,000 cells/well, n = 3 per measured variable) for up to 96 hours (37 °C, 5% CO_2_).

### Porcine IVD cell culture with CDH blocking antibody treatment

In parallel, two additional sets of cells (45,000 cells/well, n = 3 per measured variable) cultured upon PEG-LM were treated with 40 μg/mL CDH2 blocking antibody (C3865, Sigma-Aldrich) or 20 μg/mL CDH1 blocking antibody (DECMA-1, Sigma-Aldrich). Blocking antibody was added to culture media for their respective treatment groups every 24 hours for the duration of the culture period.

### Rat disc experiments

All animal protocols were approved by the local government agency (Department of Health, Hong Kong SAR) and institutional ethics committee (CULATR), and performed in accordance with relevant guidelines and regulations. The University of Hong Kong is an AAALAC accredited facility and follows guidelines of the Department of Health, Special administrative region of Hong Kong. All procedures were performed in accordance with Cap. 340 Animals (Control of Experiments) Ordinance and Regulations established by the Department of Health. Four month-old female Lewis rats were anesthetized (Ketamine:Xylazine, 2:1, 1 ml/kg i.p.). Radiographs were taken to locate caudal disc levels 4–5, 5–6, and 6–7. The tail skin was sterilized and was longitudinally incised over target disc levels. The discs were perpendicularly inserted with a 34 G gauge needle (Hamilton) at a depth of 3 mm through the anulus fibrosus, followed by injection of 2ul 200ug/ml CDH2 ectodomain antibody (Santa Cruz) or rabbit immunoglobulin G (IgG, Santa Cruz) into the nucleus pulposus. The skin and muscles were sutured and the animals were allowed to move freely in the cage after the operation. Animals were sacrificed at multiple time points from 2 to 8 weeks after injection (n = 3 per timepoint).

### Immunohistochemistry: animal experiments

At 2 and 8 weeks, spinal motion units were harvested, fixed overnight in 4% paraformaldehyde, and decalcified in Morse’s solution for paraffin embedding and sectioning. After dewaxing and rehydration, sections were treated with 0.8% hyaluronidase at 37 °C for 30 minutes and heated in citrate buffer (pH6) for 13 minutes, followed by blocking with 2% goat serum. The sections were incubated with rabbit antibodies (1:50) against CDH2 (2447–1, Epitomics, USA), type II collagen (ab34712, Abcam), type I collagen (ab34710, Abcam), aggrecan (ab36861, Abcam), β-catenin (Abcam, ab6302), T-brachyury (SC-17745, Santa Cruz), CDH1 (SC-31020, Santa Cruz) at 4 °C overnight, and then with goat anti-rabbit AlexaFlour 594 secondary antibodies (Invitrogen). The sections were mounted in Vectashield containing DAPI (Vector, USA), and visualized for fluorescence labeling (ECLIPSE 80i, Nikon). Separate sections were stained with Harris hematoxylin/eosin and visualized with brightfield microscopy to view tissue and cellular structure and organization.

### Immunohistochemistry: PEG-LM hydrogels

After the culture period, samples were fixed in 4% paraformaldehyde (Electron Microscopy Sciences, Hatfield PA) for 20 minutes, washed with DPBS, blocked for 1 hour with serum blocking solution (3.75% BSA and 5% non-immune goat serum), and immunolabeled for CDH2 (Abcam 12221, 150x overnight), CDH1 (Abcam 15148, 150× overnight), or β-catenin (Abcam 6302, 150× overnight). An appropriate isotype matched rabbit antibody was used as control (rabbit IGg, Abcam). Samples were treated with goat-anti-rabbit Alexa-Fluor 488 or 633 secondary antibody (Invitrogen) for 30 minutes at room temperature, shielded from light. Concurrently, while staining secondary antibody, other samples were immunolabeled for phalloidin (Alexa-phalloidin-488 or 633, Invitrogen) for 30 minutes at room temperature, in order to visualize actin fibers.

Cell nuclei were stained using propidium iodide (Sigma, 0.33 mg/mL) for 20 minutes at room temperature, shielded from light. Immediately after each stain, tissue sections were mounted, coverslipped, and imaged via confocal microscopy (Zeiss LSM 510, 40x magnification).

### Biochemical analysis

NP and AF cell matrix production of sulfated glycosaminoglycans (sGAG) was analyzed after culture using the dimethymethylene blue (DMMB) spectrophotometric method as previously described^45^. Media overlay from culture samples was collected while cells and hydrogels remaining in corresponding wells after media removal were digested in papain solution (125 μg/mL in DPBS with 5 mM EDTA and 5 mM cysteine, 2 hours, 65 ^○^C). Control wells were also used (hydrogels only), and processed in parallel. Absorbance (535 nm) in papain digests following reaction with DMMB was measured on a plate reader (Perkin-Elmer Enspire Multimode Reader). sGAG concentrations were determined from a standard curve prepared from chondroitin-4-sulfate (Sigma-Aldrich). For all samples, DNA content was also measured by picogreen assay (Quant-iT, Invitrogen) and total sGAG (media overlay plus cell digest) content was normalized to total DNA content.

### mRNA extraction

After the culture period, mRNA was extracted from all samples. Cells were pooled from four wells to collect sufficient mRNA for one sample. Samples were lysed by the addition of RLT buffer and β-mercaptoethanol, and TRIzol reagent (Life Technologies) was used before mRNA extraction was performed using the RNeasy mini kit plus DNase I digestion (Qiagen). mRNA integrity and concentration was verified by absorbance at 280 and 340 nm (ND-1000 Spectrophotometer, ThermoScientific). mRNA was reverse transcribed into cDNA using the iScript cDNA synthesis kit (Biorad). cDNA samples were diluted to a final concentration of 10 ng/μL using RNAse-free DNAse-free water.

### Quantitative real-time PCR (qRT-PCR)

qRT-PCR, using Taqman primer probes (Life Technologies) of porcine or human NP-specific and NP-matrix related gene-specific primers, was performed on cDNA obtained from NP and AF cells after 96 hour culture upon each of the substrates for all treatment conditions. The genes analyzed via qRT-PCR were: CDH2 (porcine: custom-designed by Life Technologies, human: Hs00983056_m1), CDH1 (human only: Hs01023894_m1), T-brachyury (porcine: Ss03374654_g1, human: Hs00610080_m1), type II collagen (porcine: Ss03373344_g1, human: Hs00156568_m1), laminin β1 (porcine: Ss03375563_u1), laminin a1 (human: Hs00300550_m1), and aggrecan (porcine: Ss03374823_m1, human: Hs00153936_m1). The housekeeping gene, 18 s (4308329, Life Technologies) or GAPDH (402869, Applied Biosystems), was used as an internal control for porcine or human samples, respectively. qRT-PCR reactions were performed in duplicate (StepOnePlus, Applied Biosystems) using standard conditions (12.5 μL 2× universal master mix, 1.25 μL Taqman primer probes, 9.25 μL ddH2O, and 2 μL 10 ng/μL cDNA). Fold-differences were calculated using 2^−ΔΔCt^, where the first Δ accounted for fold change over housekeeping gene and the second Δ accounted for fold change over stiff PEG-LM hydrogels. For human samples, the second Δ accounted for fold change over juvenile cells on stiff PEG-LM hydrogels as gene expression for many markers for degenerate human samples on stiff PEG-LM were not detectable or absent via qRT-PCR.

### β-catenin ELISA and western blot

After the culture period, protein from cells collected from 4 wells was extracted and phosphorylated β-catenin (Abcam, ab119656) were measured via ELISA (Perkin-Elmer Enspire Multimode Reader). Additionally, western blot analysis was performed to test for total β-catenin (Cell Signaling Technology, 9562) translocation from the cytoplasm to nucleus. The cytoplasmic and nuclear fractions were separated using the celLytic NuCLEAR extraction Kit (Sigma-Aldrich), and protein concentration was measured using the BCA Assay (Pierce). 5 μg of proteins for each well were separated on 10% SDS-PAGE gels and transferred to a PVDF membrane. The PVDF membrane blots were incubated overnight at 4 °C with the following primary antibodies: anti-β catenin (1: 1000, Cell signaling), anti-Lamin A (1:1000, Cell Signaling), and mouse anti-β-actin (1:1000, Sigma A5441) and anti-GAPDH (1:1000, Cell Signaling) for loading control, respectively. LI-COR IR dye conjugated specific goat anti-rabbit, and goat anti-mouse IgG secondary antibody (1:10,000) was added and incubated for 45 minutes at room temperature. Immunoblots were imaged and analyzed using the Odyssey imaging system (LI-COR Biotechnology, Lincoln, NE).

### Statistical Analysis

All statistical analyses were performed using JMP software (SAS). Significance was tested at p < 0.05 unless otherwise noted.

#### For porcine NP and AF studies

A two-way ANOVA with Tukey’s post-hoc analysis was used to analyze differences in matrix production in NP and AF cells (substrate, cell type). When IVD cells were treated with CDH blocking antibody or CRISPR knockdown, a two-way ANOVA with Tukey’s post-hoc analysis was performed within each cell type so that significant differences in matrix production due to treatment and substrate could be analyzed. Statistical analyses were also performed for qRT-PCR findings: within each gene analyzed, a two-way ANOVA with Tukey’s post-hoc analysis was performed to compare differences between cell type and substrate, and for blocking antibody or knockdown studies, each cell type was analyzed separately to compare substrate and treatment.

#### For human NP and AF studies

Before statistical analyses were performed, adult, degenerate human NP cells were separated into two categories, CDH2+ and CDH2- samples. Cells from human subjects were considered to be CDH2+ if after culture on PEG-LM, more than 90% of immunolabeled cells were positive for CDH2 (>90%, analyzed via IMAGEJ); otherwise cells from the human sample isolation were considered to be CDH2-.

#### NP and AF cells were analyzed separately

A two-way ANOVA (cell, substrate) with Tukey’s post-hoc analysis was used to test for differences in sGAG production amongst cells after periods of culture. A two-way ANOVA (cell, substrate) with Tukey’s post-hoc test was performed for each value of 2 ^−ΔΔCt^ analyzed.

## Additional Information

**How to cite this article**: Hwang, P. Y. *et al*. N-cadherin is Key to Expression of the Nucleus Pulposus Cell Phenotype under Selective Substrate Culture Conditions. *Sci. Rep*. **6**, 28038; doi: 10.1038/srep28038 (2016).

## Supplementary Material

Supplementary Information

## Figures and Tables

**Figure 1:**
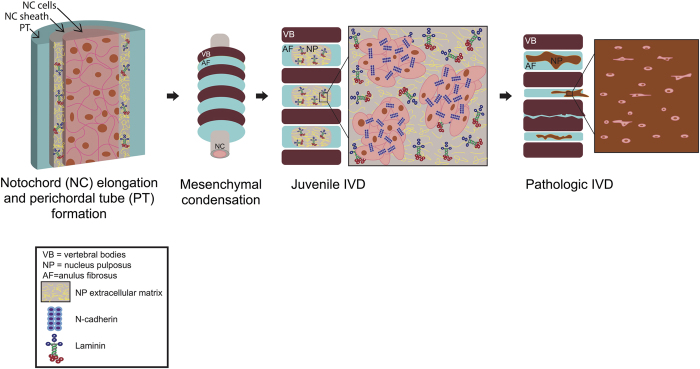
Schematic of intervertebral disc development and degeneration. Healthy, juvenile IVD is characterized by NP cells existing in CDH2 positive cell clusters in a laminin-rich, soft matrix environment, which undergoes dramatic changes with aging or degeneration.
