# Supplementary material for: N-cadherin is Key to Expression of the Nucleus Pulposus Cell Phenotype under Selective Substrate Culture Conditions
Source: Sci Rep. 2016 Jun 13;6:28038. doi: 10.1038/srep28038 (PMC4904275; doi:10.1038/srep28038)
Supplement: Supplementary Information [file srep28038-s1.pdf]

**N-cadherin is Key to Expression of the Nucleus Pulposus Cell Phenotype under Selective Substrate Culture Conditions**

Priscilla Y Hwang[1,2]

Liufang Jing[1, 2]

J Chen[3]

Foon-Lian Lim[4]

Ruhang Tang[5]

Hyowon Choi[6]

Kenneth M Cheung[4]

Makarand Risbud[6]

Charles A Gersbach[1]

Farshid Guilak[1,2,5]

Victor Y Leung[4]

LA Setton*[1,2,5]

[1] Department of Biomedical Engineering, Duke University, Durham NC 27713

[2] Department of Biomedical Engineering, Washington University in St. Louis, St. Louis Missouri 63130

[3] Department of Orthopaedic Surgery, Duke University Medical Center, Durham NC 27713

[4] Departments of Orthopaedics & Traumatology, The University of Hong Kong, Hong Kong SAR, China

[5] Department of Orthopaedic Surgery, Washington University in St. Louis, St. Louis Missouri 63110

[6] Department of Orthopaedic Surgery, Thomas Jefferson University, Philadelphia PA

**Keywords:** CDH2, CDH1, intervertebral disc, nucleus pulposus, annulus fibrosus, cell-cell interactions, laminin, polyethylene glycol, CRISPR, notochord, brachyury, lamin

**Supplemental Figure 1: Characterization of PEG-LM hydrogels: (A)** Synthesis of PEG-LM (adapted from Francisco et al 2013, Biomaterials) **(B)** Percent of laminin coating on hydrogel surface as measured in IMAGEJ **(C)** Uniform pegylated laminin coated on PEG-LM hydrogel surface (representative images displayed) **(D)** Shear modulus of PEG-LM hydrogels formulized as measured via rheometry (adapted from Francisco et al 2013, Biomaterials)

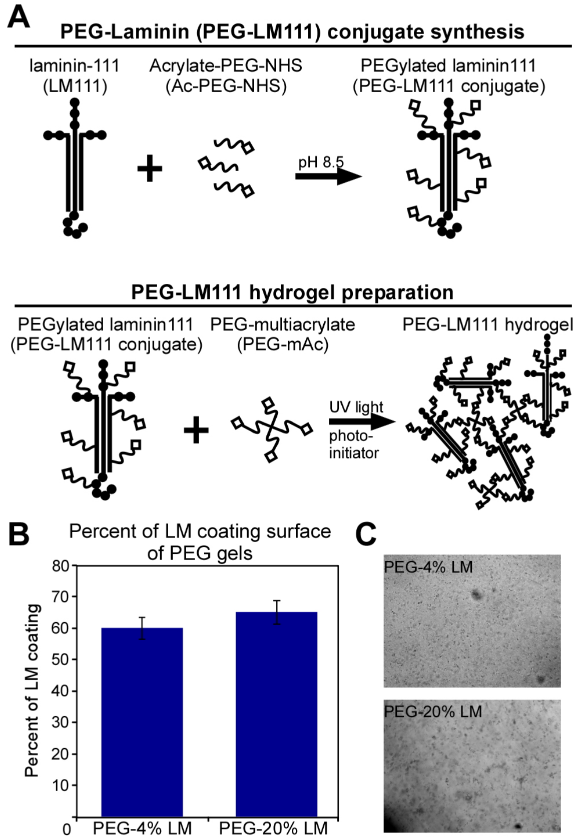


D

| | PEG-4% LM | PEG-20% LM |
|---|---|---|
| Shear Modulus | 0.3 kPa | 29 kPa |

**Supplemental Figure 2: Porcine and human AF cells do not form cell clusters on soft, laminin (LM) hydrogels and do not express high levels of juvenile NP markers (A)** Representative immunostaining of phalloidin and cadherins for porcine AF cells on soft and stiff PEG-LM (green = protein, red = cell nuclei, scale bar = 50 mm) **(B)** Changes in sGAG production for porcine AF cells on soft and stiff PEG-LM **(C)** Quantification of gene expression for juvenile NP cell phenotype markers in porcine AF cells on soft, relative to AF cells on stiff (CDH2 = N-cadherin, T= brachyury, LM1= Laminin1, AGC = aggrecan, COL2 = type II collagen) **(D)** Representative immunostaining of phalloidin and cadherins for juvenile (juv) and degenerate (deg) human AF cells on soft PEG-LM **(E)** Same as C but with human AF cells on PEG-LM **(F)** Same as D but with human AF cells on PEG-LM (additional marker CDH1 was quantified in human)

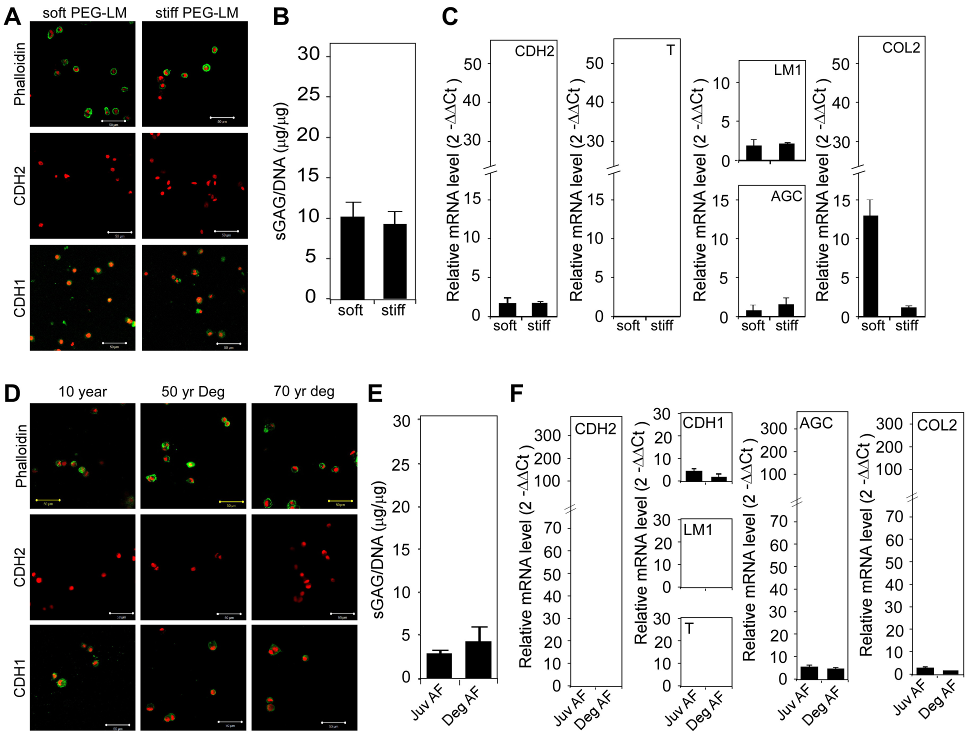

**Supplemental Figure 3: Matrix synthesis and gene expression of NP markers are not affected by CDH blocking antibody treatment in porcine AF cells (A)** Changes in sGAG production for porcine AF cells on soft and stiff PEG-LM after blocking antibody treatment **(B)** Quantification of gene expression for juvenile NP cell phenotype markers in porcine AF cells on soft, relative to AF cells on stiff after blocking antibody treatment (2-way ANOVA with Tukey's post-hoc analysis, *p<0.05) (Key: 1= No treatment conditon, 2= CDH2 blocking antibody treatment, 3 = CDH1 blocking antibody treatment; CDH2 = N-cadherin, T= brachyury, LM1= Laminin1, AGC = aggrecan, COL2 = type II collagen)

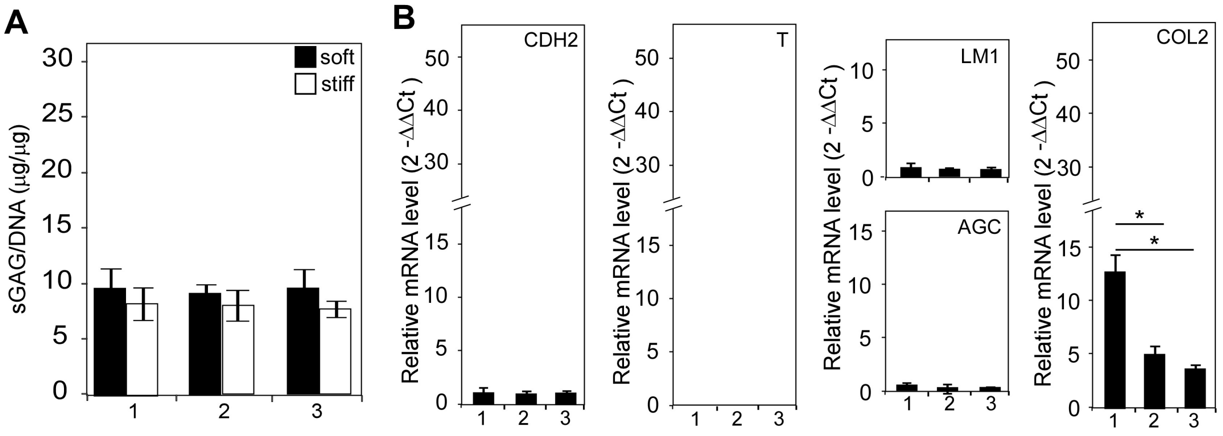

**Figure 4:** Images of full western blot samples for β-catenin, LaminA/C, and their respective housekeeping markers (from Figure 2)

**Western Blot Images for Figure 2G**

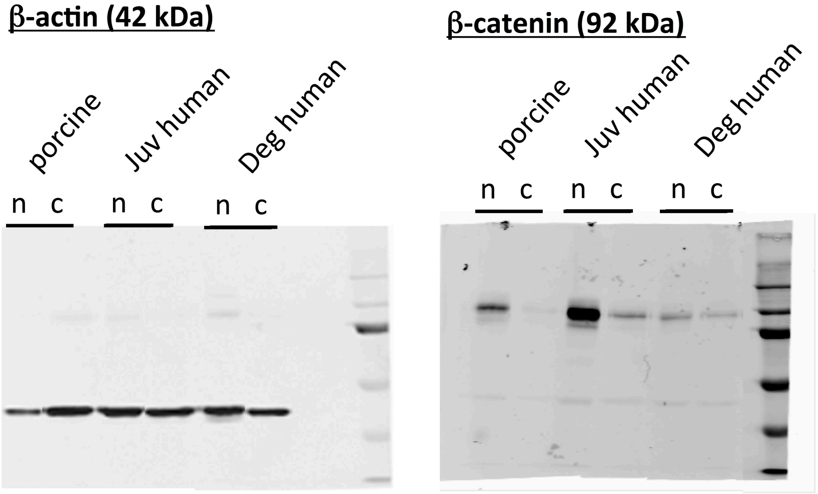


**Western Blot Images for Figure 2H**

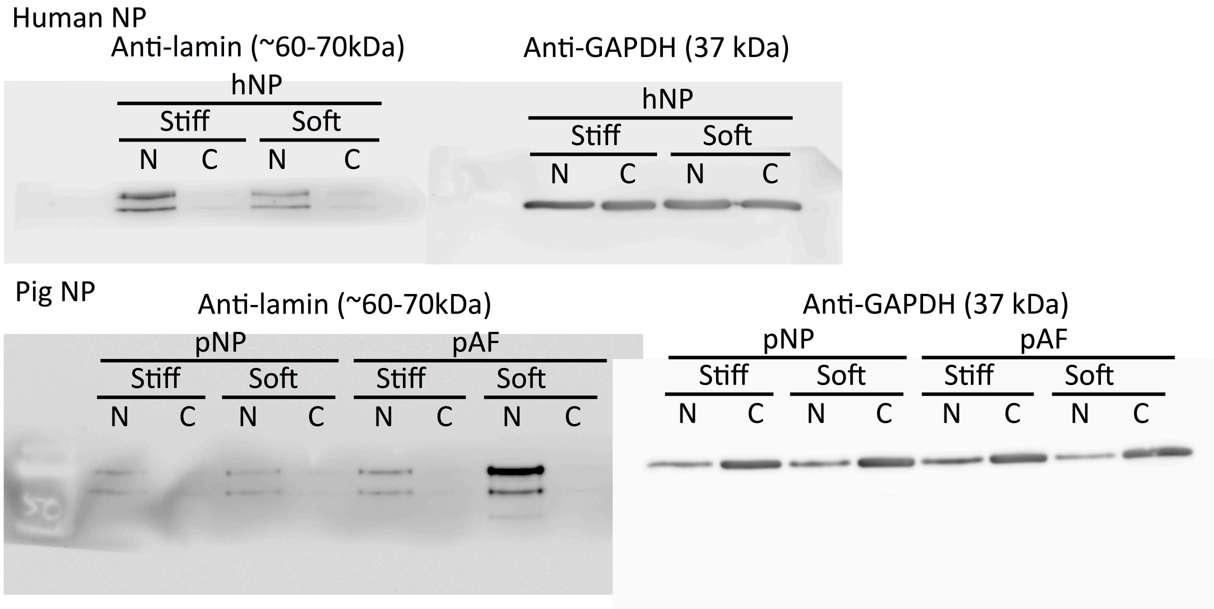

**Supplemental Table 1:** Information regarding human samples analyzed and separated into groups (F= Female, M=Male, C= Caucasian, AA=African American, + = N-cadherin positive, - = N-cadherin negative)

| | Sample # | Age | Sex | Race | CDH2 (+/-) |
|---|---|---|---|---|---|
| Juvenile | 1 | 9 | F | C | + |
| | 2 | 10 | M | C | + |
| | 3 | 7 | M | C | + |
| | 4 | 13 | M | C | + |
| Degenerate | 5 | 67 | M | C | + |
| | 6 | 79 | F | C | - |
| | 7 | 67 | F | C | - |
| | 8 | 63 | F | AA | - |
| | 9 | 66 | M | C | - |
| | 10 | 69 | M | C | + |
| | 11 | 75 | M | C | + |
| | 12 | 65 | M | AA | + |
| | 13 | 73 | F | C | - |
| | 14 | 70 | M | AA | + |
| | 15 | 62 | F | C | - |
| | 16 | 60 | M | C | - |
| | 17 | 68 | F | C | + |
| | 18 | 48 | F | C | + |
| | 19 | 53 | M | AA | - |
| | 20 | 63 | M | C | - |
| | 21 | 50 | M | C | + |
| | 22 | 35 | F | C | + |
